# Clinical Significance of Lipid Transport Function of ABC Transporters in the Innate Immune System

**DOI:** 10.3390/membranes12111083

**Published:** 2022-10-31

**Authors:** Stanislav Kotlyarov, Anna Kotlyarova

**Affiliations:** 1 Department of Nursing, Ryazan State Medical University, 390026 Ryazan, Russia; 2 Department of Pharmacy Management and Economics, Ryazan State Medical University, 390026 Ryazan, Russia

**Keywords:** ABC transporters, lipids, innate immune system, ABCA1, ABCB1, lipopolysaccharide

## Abstract

ABC transporters are a large family of proteins that transport a variety of substrates across cell plasma membranes. Because of this, they are involved in many physiological processes. It is of interest to note that many ABC transporters are involved in the transport of various lipids. In addition, this function may be related to the innate immune system. The evidence that ABC transporters are involved in the regulation of the innate immune system through the transport of various substances greatly enhances the understanding of their clinical significance. ABC transporters are involved in the cellular homeostasis of cholesterol as well as in the regulation of its content in lipid rafts. Through these mechanisms, they can regulate the function of membrane proteins, including receptors of the innate immune system. By regulating lipid transport, some members of ABC transporters are involved in phagocytosis. In addition, ABC transporters are involved in the transport of lipopolysaccharide, lipid mediators of inflammation, and perform other functions in the innate immune system.

## 1. Introduction

The ATP-binding cassette transporters (ABC transporters) are a family of proteins that transport chemically different substrates through the lipid bilayer of cell membranes, at the expense of the energy obtained by ATP hydrolysis. Currently, 48 human ABC transporters have been described, which are divided into seven subfamilies, known as ABCA to ABCG, based on their structural organization. ABC transporters contain two conserved nucleotide-binding domains (NBD), which convert the energy of ATP binding and hydrolysis into conformational changes of two transmembrane domains (TMD) that are involved in substrate recognition [1,2].

Due to the chemical diversity of substrates, ABC transporters are widely involved in various biological processes [3]. Members of the ABCA and ABCG subfamilies are well known for their role in the transport of various lipids, which allows them to be considered as important players in many clinically relevant diseases, such as atherosclerosis. In turn, members of the ABCG and ABCC subfamilies are involved in the mechanisms of multidrug resistance, which is of great clinical importance and is the subject of numerous studies.

**Figure 1:**
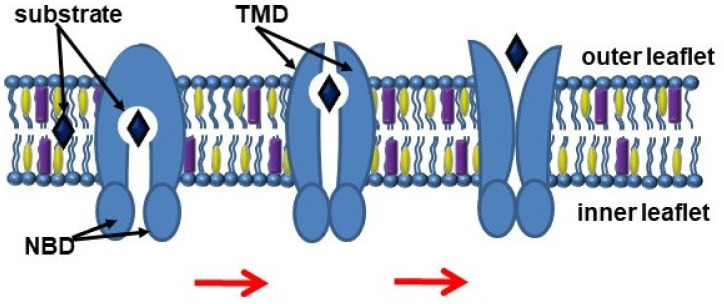
Scheme of the ABC transporter function. Abbreviations: NBD, nucleotide-binding domains; TMD, transmembrane domains.

The export of substrates by ABC transporters is described through the “alternating access” model, which involves switching between inward and outward facing transmembrane cavity (Figure 1) [4]. NBDs dimerize and induce a switch of TMDs from an inward-facing conformation to an outward-facing conformation, thereby moving the substrate [5]. In turn, TMDs form a transport substrate-binding cavity, which can open into either the cytoplasmic or exofacial leaflet of the plasma membranes [6].

Much of the data forming the current paradigm of ABC transporter function were obtained by studying bacterial MsbA. Its function is related to the transport of lipopolysaccharide (LPS), which is a major lipid component of the outer membrane leaflet of most Gram-negative bacteria and plays an important role in cell membrane formation [7]. MsbA is located in the inner membrane of Gram-negative bacteria and performs the first stage of LPS transport, moving LPS from its synthesis site on the inner leaflet of the inner membrane to the outer leaflet of the inner membrane [8,9,10,11]. LPS is then further modified and transported to the outer leaflet of the outer membrane by the Lpt transport mechanism [12,13]. The structure of LPS includes a lipid A moiety, a core oligosaccharide and a long-chain O-antigen polysaccharide [14]. Lipid A is the conserved part of LPS and induces the innate immune response during bacterial infection [15,16].

MsbA is considered to be a homologue of the human ABCB1 transporter, with which it partially shares its substrate spectrum. At the same time, the MsbA-mediated transport mechanism has features similar to those observed in the structure of the TLR4-MD-2-LPS complex [17], which suggests some commonality of strategies for LPS recognition [7].

There is a growing body of evidence that supports the involvement of ABC transporters in the mechanisms of the innate immune system. This involvement is related to the transport function of the ABC transporters. The purpose of this review is to discuss the involvement of ABC transporters in innate immune system mechanisms through their lipid transport function.

## 2. The Innate Immune System

The innate immune system is an evolutionarily ancient defense system that allows the constancy of the macromolecular composition of the body. It provides resistance to infectious agents through detection and removal of foreign molecules [18]. The innate immune system relies on a large number of different cellular and humoral mechanisms to perform its functions [19]. A growing body of evidence strengthens the understanding of the significance of the cross-links between lipid metabolism and the innate immune system. Lipid mediators derived from fatty acids are involved in inflammation in various ways. On the one hand, leukotrienes (LT) contribute to inflammation, whereas specialized pro-resolving mediators, on the contrary, contribute to the resolution of inflammation.

Macrophages are important participants in the innate immune system. These cells show different roles in inflammation. On the one hand, M1 polarization is considered pro-inflammatory; on the other hand, the M2 phenotype promotes the resolution of inflammation. Macrophage polarization is characterized by metabolic changes in the cells. Interestingly, cholesterol loading of macrophages contributes to their proinflammatory activity, which is characterized by the production of inflammatory mediators such as cytokines and reactive oxygen species, which attract other cells and contribute to increased inflammation [20,21].

Of interest is the evidence that ABCB1 (MDR1, P-glycoprotein) function and expression are higher in M2 macrophages [22]. These differences may influence intracellular drug concentrations between these two cell subsets. In another study, ABCB1 expression levels were elevated in M2a subtype macrophages, which participate in the role of phospholipids in phagocytosis and retinoic acid-mediated signal transduction [23]. In addition, ABCC1 (MRP1) was shown to be upregulated in M1 macrophages, whereas ABCG2 (BCRP) expression was upregulated in M2 polarized macrophages [24]. ABCA1 expression was also shown to be downregulated in M1 macrophages compared with M0 and M2 [25]. In another study, obese adipose tissue macrophages showed increased cholesterol export and expression of ABCA1 and ABCG1 compared with lean adipose tissue macrophages. Meanwhile, cholesterol export and ABCA1 expression are suppressed in M1 polarized macrophages through the JAK/STAT pathway [26]. In addition, Abcg1 is expressed predominantly by M2 macrophages, and loss of Abcg1 modulates the number of M2 macrophages and the ratio of M1 to M2 adipose tissue macrophages compared with the number found in wild-type mice. ABCG1 plays an important role in the metabolism of intracellular sterols in M2 adipose tissue macrophages [27]. Thus, cholesterol export from M2 macrophages by phagocytosis is an important mechanism supported by ABC transporters.

As noted previously, LPS is an important component of the outer membrane of Gram-negative bacteria, participating in the protection of bacteria from adverse environmental conditions, as well as in providing resistance to drugs [28]. LPS detection in humans is carried out by the TLR4 receptor, which belongs to the Toll-like receptor family. These receptors allow the innate immune system to detect various molecular patterns associated with the pathogen and molecular patterns associated with damage. Moreover, TLRs are not only involved in the antimicrobial defense of the body, but are also involved in the pathogenesis of various noninfectious inflammatory diseases, including atherosclerosis [29].

TLR signaling involves the binding of ligands to the region of extracellular leucine-rich repeats, leading to oligomerization of intracellular TIR receptor domains, which in turn activate adaptor molecules such as MyD88, TIRAP, TRIF, TRAM, and SARM for subsequent signal transduction from the membrane surface to the cytosol [30,31,32,33].

According to its biological role, TLR4 is expressed by cells involved in the body’s first-line defense against pathogens, including neutrophils, macrophages, and dendritic cells [34].

Interestingly, TLR4 is directly related to cholesterol metabolism. TLR4 can affect cholesterol metabolism in macrophages. Bacterial and viral components acting through TLR3 and TLR4 receptors have been shown to decrease the ability of macrophages to export excess cholesterol through inhibition of LXR target gene expression [20]. Moreover, the ability of microbial agents to inhibit LXR function is mediated through MyD88-independent mechanisms of the TLR3/4 signaling pathway. Expression and activation of IRF3 in macrophages promote inhibition of LXR transcriptional activity at the ABCA1 promoter, which inhibits cholesterol efflux from macrophages [20].

In this regard, it is worth noting the fact that TLR activation is associated with cellular cholesterol levels and is regulated by reverse cholesterol transport. ABC transporter knockdown enhances signaling through TLR4 and MyD88/TRIF in macrophages [35].

Another proinflammatory mechanism related to cholesterol is mediated by activation of the NLRP3 inflammasome. It is known that cholesterol crystals can act as danger signals, causing neutrophils to release neutrophil extracellular traps (NETs) that will stimulate macrophages to release cytokines IL-1β and IL-18 through activation of several TLRs [36,37]. The proinflammatory cytokines IL-1β and IL-18 promote further formation of NETs, thus forming a vicious circle [37]. Cholesterol accumulation in dendritic cells due to ABCA1 and ABCG1 deficiency promotes NLRP3 inflammasome activation [38]. *Abca1/Abcag1* deficiency also enhances the accumulation of free and esterified cholesterol in spleen neutrophils [39].

Thus, many members of ABC transporters demonstrate involvement in innate immunity, which is of clinical interest.

## 3. ABC Transporters

### 3.1. The ABCA Subfamily

**Figure 2:**
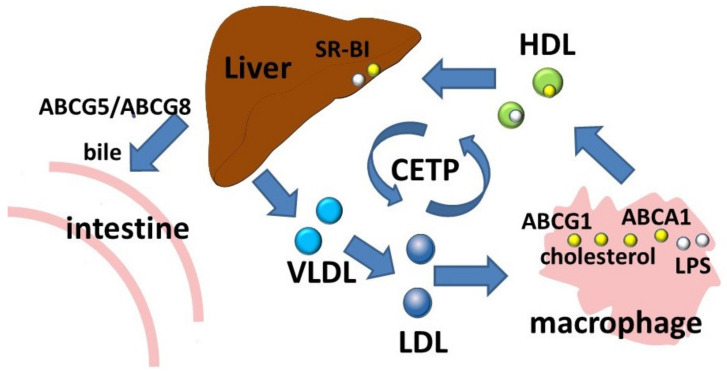
Reverse cholesterol transport and LPS transport. Abbreviations: ABCA1, ATP-binding cassette subfamily A member 1; ABCG1, ATP-binding cassette subfamily G member 1; CETP, cholesteryl ester transfer protein; HDL, high density lipoprotein; LDL, low density lipoprotein; LPS, lipopolysaccharide; SR-BI, scavenger receptor class B type 1; VLDL, very low density lipoprotein.

The ABCA subfamily consists of 12 members which are involved in the transport of lipid molecules across membranes [40]. Members of this subfamily are well known for their role in lipid transport [3]. ABCA1 is considered to be a key participant in reverse cholesterol transport, a process by which cholesterol moves from cells to the extracellular acceptor to form the resulting HDLs (Figure 2). Macrophages, as a result of reverse cholesterol transport, maintain an optimal balance of cholesterol, the excessive accumulation of which has proinflammatory effects. Absence of ABCA1 leads to significant changes in the morphology, properties, and functional activity of macrophages [41]. In addition to free (non-esterified) cholesterol transport, ABCA1 functions as a membrane phospholipid translocase, and its enzymatic activity leads to the transfer of phospholipid molecules from the cytoplasmic leaflet to the outer leaflet of the cellular plasma membrane [6].

ABCA1 has been shown to exert an anti-inflammatory effect by promoting cholesterol efflux followed by attenuation of signal transduction via Toll-like receptors [35]. At the same time, macrophages *Abca1^−/−^ Abcg1^−/−^* have shown enhanced inflammatory gene responses to TLR2, TLR3, and TLR4 ligands [26,35]. In turn, *Abca1^−/−^ and Abcg1^−/−^* macrophages were shown to have increased lipid rafts and produced higher levels of tumor necrosis factor alpha (TNFα) and Interleukin (IL)-6 after LPS stimulation compared with wild-type macrophages [35]. Thus, the involvement of ABCA1 in TLR4 regulation may be through changes in cholesterol content in the lipid rafts of the plasma membrane, into which signaling molecules such as TLR4 are recruited.

ABCA1 predominantly localizes in the plasma membrane of the cell, which requires palmitoylation of the protein [42,43,44,45]. ABCA1 activity leads to the reorganization of lipids in the plasma membrane, which is characterized by redistribution of membrane cholesterol from raft domains to non-raft domains [46,47]. At the same time, ABCA1 itself can be located either in the non-raft domains [47,48,49] or in the raft domains [6,50]. Interestingly, ceramide enhances cholesterol efflux into apolipoprotein A-I by increasing the presence of ABCA1 on the cell surface [51].

Cholesterol is involved in maintaining the spatial structure of the plasma membrane. This is related to the chemical structure of the cholesterol molecule and its spatial arrangement in the plasma membrane. Cholesterol is an important component of lipid rafts, and changes in cholesterol content in the plasma membrane affect its structure and function. Cholesterol can influence the biophysical properties of the membrane and can also interact directly with specific protein sites, so that it can participate in the regulation of the function of membrane proteins [52].

Proteins that interact with cholesterol are thought to have specific amino acid sequences that play a role in this interaction [53]. A known amino acid sequence is the cholesterol-binding domain (CRAC, Cholesterol Recognition/interaction Amino acid Consensus sequence), which has been identified in proteins that interact with or are regulated by cholesterol [54,55,56,57]. The CRAC amino acid sequence includes the following set of amino acids: L/V–X (1–5) –Y–X (1–5) –R/K (with X = any amino acid) [56]. The other sequence, the CARC motif, has similar properties in binding to transmembrane proteins and has the reverse amino acid sequence: R/K–X (1–5) –Y/F–X (1–5) –L/V, (with X = any amino acid and tyrosine can be replaced with phenylalanine). Both CRAC and CARC sequences have been found in the transmembrane domain of the TLR4 receptor, providing a putative link between cholesterol and the regulation of receptor signal transduction [53].

ABCA1 can alter the cholesterol content of macrophage plasma membranes, which affects the stability of lipid rafts and can regulate TLR4 activity [58]. Deletion of *ABCA1* leads to increased localization of TLR4 in lipid rafts in both resting and stimulated states [58]. *Abca1*-deficient mice have increased circulating levels of chemokines, cytokines, and growth factors. In addition, *Abca1*-deficient macrophages contain more cellular cholesterol ester, which corresponds to higher expression of scavenger receptors and greater TNFα secretion in response to LPS [41]. In turn, ABCA1 overexpression leads to a significant redistribution of cholesterol and sphingomyelin from lipid rafts to non-raft membrane regions, which can degrade membrane lipid rafts and enhance cholesterol efflux into ApoA-I [46].

In turn, TLR4 activation can inhibit ABCA1 expression, reducing the macrophage cholesterol efflux [20,59].

Decreased transport function of ABCA1 leads to excessive intracellular accumulation of cholesterol in macrophages, which contributes to the initiation of NLRP3 inflammation. Cholesterol accumulation in *Abca1/Abcg1*-deficient myeloid cells has been shown to activate the NLRP3 inflammasome, which promotes neutrophil infiltration and netosis in atherosclerotic lesions [39].

The ABCA1 transporter has also been shown to be involved in the removal of an immunostimulatory bacterial lipid, LPS (Figure 2) [60,61]. In experiments with preconditioning of *ABCA1*-deficient macrophages with LPS, a significant decrease in LPS outflow was observed, resulting in prolonged persistence of LPS on the cell surface [61].

ABCA1 is an important subject for studies on the pathogenesis of atherosclerosis. ABCA1 mutations are associated with the development of Tangier disease, which is characterized by a significant decrease in HDL levels, which corresponds to premature atherosclerosis [62]. Decreased ABCA1 protein has been shown to play a key role in the pathogenesis of carotid atherosclerosis [63]. In one study, ABCA1 protein expression was significantly decreased in plaques compared with control tissues [64]. Moreover, ABCA1 may serve as an important marker of plaque instability [63]. Leukocyte ABCA1 has been shown to play an important role in protecting against atherosclerosis, whereas more widespread and larger atherosclerosis lesions develop in the absence of ABCA1 leukocyte [65]. These and other data suggest that ABCA1 is a promising target for drug action to prevent and treat atherosclerosis [66].

Another member of the subfamily, ABCA7 is involved in cross-links between fatty acid metabolism and inflammation in the brain. Disturbances in these connections may contribute to the development of Alzheimer’s disease [67]. In addition, several studies have shown possible involvement of ABCA7 in phagocytosis [68,69]. ABCA7 is expressed in macrophages [68,70,71], where it can be localized in the plasma membrane, or intracellularly [72,73,74,75]. The putative involvement of ABCA7 in phagocytosis is due to the fact that the protein has high amino acid sequence similarity with CED-7, which is required for efficient phagocytosis of apoptotic cells [69]. The CED-7 protein in the nematode *Caenorhabditis elegans* functions in phagocytic and apoptotic cells during phagocytosis and is required for the clustering of CED-1, a transmembrane receptor that initiates uptake signals. In doing so, CED-7 carries out the exposure of phospholipid ligands on the surface of apoptotic cells. The ABCA5 protein is also involved in cholesterol transport, and ABCA5 expression has been shown to increase in monocytes and macrophages after incubation with acetylated LDL [76,77].

Thus, members of the ABCA subfamily play a significant role in the mechanisms of innate immunity mediated by lipid transport, which is of great clinical importance [78,79].

### 3.2. The ABCB Subfamily

ABCB1 is the most well-characterized member of the ABCB subfamily. The ABCB1 (MDR1, P-glycoprotein) protein encoded by the *ABCB1* gene is expressed by various immune cells where it may be involved in migration, differentiation, survival, or cytotoxic function [80].

This transporter is well known for its role in multidrug resistance due to its ability to efflux a variety of molecules with different chemical structures (cyclic, linear, polar, non-polar, linear-hydrophobic, aromatic) and different molecular weights (from 250 to 4000 Da) [81]. ABCB1 has a wide substrate specificity, which allows it to transport chemically diverse molecules, including drugs [82,83,84].

ABCB1 has been shown to transport lipophilic and amphipathic compounds that accumulate in the lipid bilayer, lining up in the interphase region between the lipid head group and the first few carbon atoms of the lipid acyl chains [85]. Given the knowledge that ABCB1 removes drugs directly from the membrane rather than from the aqueous phase [83], it is assumed that the transporter is a hydrophobic “vacuum cleaner” responsible for removing potentially harmful lipophilic compounds from the membrane [81,86].

The capture of ABCB1 drugs inside the lipid membrane can occur due to weak electrostatic interactions between hydrogen bond acceptor groups, including phenyl rings and tryptophans (i.e., π-electron donor systems), in drugs and hydrogen bond donor groups (i.e., π-electron acceptor systems) in the transmembrane region of ABCB1 [87]. Whether a drug can be classified as a substrate for ABCB1 depends on the cross-sectional area of the drug as well as on the lipid composition of the plasma membrane, which determines the lateral density of lipid packing [88,89]. It is important to note that lipid packing density is affected by membrane composition, including cholesterol content [87].

In addition to xenobiotic transport, ABCB1 performs many other physiologically relevant functions, such as being involved in lipid transport [82,86,90], moving lipids from the inside to the outside of the cell plasma membrane [53,82]. Cholesterol is also transported by ABCB1 [91]. Interestingly, the cholesterol content in the plasma membrane can regulate the functional activity of the transporter [92,93,94,95]. This may be due to the direct interaction of ABCB1 with lipid molecules [53,82,96].

Links between ABCB1 and innate immunity support the information that TLR4 ligands and inhibitors modulate ABCB1 activity, suggesting that interaction with TLR4 is important for ABCB1 function. The innate immune inflammatory response may play a role in the ocular trabecular meshwork through modulation of ABCB1. The functional activity of ABCB1 is influenced by TLR4 agonists, suggesting that TLR4 modulation is important for ABCB1 function [97].

ABCB1 and TLR show partially overlapping substrate specificity, as TLRs can recognize some substances that have the same recognition patterns as the allocrites for ABCB1 [87]. Meanwhile, ABCB1 binds cationic and electrically neutral compounds, including nucleosides, whereas PRRs rather recognize anionic nucleotides and are activated by bacterial lipid A surrounded by anionic phosphate groups [87].

TLR2 is thought to act as a central regulator of xenobiotic defense through ABCB1. TLR2 has been shown to modulate the synthesis and activity of ABCB1 in mouse and human myeloid cells by stimulating xenobiotic efflux, which reduces cytotoxicity. Activation of ABCB1 transcription involves the MKK3/6 → p38 MAPK pathway [98], which is common to TLR2 signaling [99]. This mechanism allows the innate immune system to respond to harmful substances and carry out their immediate elimination [100].

ABC/MXR transporters are known to play a role in LPS efflux [101]. It has been shown that LPS uptake by the cells of the gastrointestinal tract can be excreted by ABCB1. Abcb4 efflux of LPS in fish back into the intestinal lumen may represent a protective mechanism to limit systemic LPS uptake and inflammation [101].

In addition to these mechanisms, ABCB1 has other extensive connections to the immune system. ABCB1 has been shown to be important for the secretion of proinflammatory cytokines such as TNFα and IFN-gamma [102]. ABCB1 is thought to mediate dendritic cell maturation and T-cell responses by influencing TNFα and IFN-γ production, thereby regulating immune responses [102]. Interestingly, ABCB1 is important for the complete activation of the response to type I interferon induced by *Listeria monocytogenes* bacteria. Moreover, inhibition of ABCB1 function by verapamil or inhibition of its transcription using mRNA silencing reduced the magnitude of the type I response in infected cells, i.e., decreased IFN release, which shows the importance of ABCB1 for the proper development of the innate immune response against intracellular pathogens [103]. In addition, R(+) verapamil improved the survival of mice that received a lethal dose of LPS by inhibiting ABCB1, which was accompanied by decreased TNFα and IFN-gamma levels and higher levels of IL-6 [104].

In addition to IFN-gamma and TNFα, ABCB1, may be involved in the release of cytokines such as IL-2 and IL-4. In addition, ABCB1 is involved in IL-12-dependent differentiation of monocytes in the dendritic cell line during antigen-presenting cell maturation. Because of this, ABCB1 regulates the ability of myeloid-derived antigen-presenting cells to elicit alloimmune Th1 responses [105]. Specific ABCB1 blockade inhibits IL-12 secretion and activation of human alloimmune T cells in vitro [106]. ABCB1 is involved in dendritic cell maturation when exposed to LPS and hypoxia, whereas inhibition of ABCB1 impaired dendritic cell maturation and reduced alloimmune T-cell proliferation [107]. ABCB1 is also involved in the regulation of leukocyte movement, especially of dendritic cells from tissues through lymphatic channels [108]. Given that dendritic cells are the link between innate and adaptive immunological responses, the importance of ABCB1 is of increasing interest.

ABCB1 is also known to secrete platelet-activating factor (PAF) [109]. PAF is a lipid mediator that mediates inflammation and is produced in response to various stimuli, including lipopolysaccharide, tumor necrosis factor, interleukin-1, activated complement, and angiotensin II [110].

Overall, these data suggest an important role for ABCB1 in innate immunity [87]. At the same time, ABCB1 inhibitors such as verapamil can impair the regulation of the immune response. This is supported by reports of the development of colitis in *mdr1a*-deficient mice. The intestinal inflammation seen in *mdr1a*^−/−^ mice results from a defect in the intestinal epithelial barrier and is characterized by dysregulation of epithelial cell growth and leukocytic infiltration into the lamina propria of the large intestine, and is consistent with signs of ulcerative colitis in humans [111]. Interestingly, colitis in these animals can be prevented by oral treatment with broad-spectrum antibiotics to kill intestinal bacteria. Even after the inflammation has ceased, a large number of CD3+ T cells remain in the intestinal mucosa [111].

Subsequent studies have greatly expanded the understanding of the role of ABCB1 in gut immunology. The gut microbiome is known to contribute to gut health and is also involved in the regulation of other organs, such as the lungs, through the production of some biologically active substances. The gut microbiome has evolved with the host, so the structure of the gut microbiome is closely related to dietary patterns, which is of great clinical importance. The gut microbiome and the ABCB1 of the gut epithelium have been found to be jointly involved in maintaining gut health. Bacterial populations containing the classes Bacilli and Clostridia contribute to the induction of ABCB1 expression in the colon by acting through butyrate and secondary bile acids produced by these bacteria from food substrates [112,113]. Butyrate, which is a short-chain fatty acid (SCFA), increases ABCB1 expression in colonic epithelial cell lines [112,114]. In patients with ulcerative colitis, there is a decrease in the number of butyrate-producing bacteria such as *Roseburia hominis* and *Faecalibacterium prausnitzii* and a decrease in the concentration of secondary bile acids in the intestine [112,115,116,117].

In addition, lithocholic acid (LCA), deoxycholic acid (DCA), and ursodeoxycholic acid (UDCA), secondary bile acids produced by intestinal bacteria, including representatives of Clostridia and Bacilli, by deconjugation and conversion of primary bile acids, enhance the induction of ABCB1 protein expression [113,118]. Thus, the gut microbiome and diet play an important role in maintaining gut health through ABCB1. These findings are supported by evidence that colitis was preceded by altered gut bacterial composition, and a high-fat diet increased the frequency and severity of colitis in Abcb1 KO mice without specific pathogens [119,120].

Lower levels of ABCB1 in the colon have been shown in patients with active inflammation and ulcerative colitis compared with controls [119,121,122]. Moreover, low levels of ABCB1 in the colon may precede malignancy [123]. Thus, ABCB1, diet, and intestinal bacteria mutually interact in colonic inflammation, which is of great clinical importance [119].

Another function of ABCB1 in gut immunology is related to the regulation of neutrophil infiltration. ABCC2 (MRP2) has been found to regulate transepithelial neutrophil migration by apical release of hepoxylin A3 (HXA3), which is a potent chemoattractant [124]. At the same time, intestinal ABCB1 exports N-acyl ethanolamine-type (NAE) endocannabinoids (eCB), which can inhibit this transepithelial migration through eCB interaction with neutrophilic cannabinoid receptor 2 (CB2), which is an important mechanism for the homeostasis of neutrophil function regulation in the gut [112,118].

Information on the protective role of ABCB1, which is expressed on intestinal epithelial cells against *Listeria monocytogenes*, is of interest [125,126]. Caco-2/MDR cells overexpressing ABCB1 have been shown to be characterized by resistance to *L. monocytogenes* invasion, whereas inhibition of ABCB1 resulted in increased invasion [125]. Meanwhile, *Salmonella enterica* serovar *Typhimurium* modulates, which is a facultative intracellular pathogen, specifically suppresses ABCB1 function, thereby increasing its ability to invade [127]. It has been found that the effector *S. Typhimurium* effector protein, SipA, modulates ABCB1 activity through a pathway involving caspase-3 [128]. These properties of *Salmonella enterica* are of great clinical interest as one of the promising areas of anti-tumor therapy [129]. *Pseudomonas aeruginosa* secreted the toxin Cif (Cystic fibrosis transmembrane conductance regulator (CFTR) inhibitory factor), which selectively reduces the expression of ABCB1 on the apical membrane in renal, respiratory, and intestinal epithelial cells [130]. These and other data confirm the protective role of the ABCB1 transporter in protecting the host not only against xenobiotics, but also against bacterial pathogens.

It is of interest to know that ABCB1 expression is a distinctive feature of mature naive B-cells because it increases during final differentiation from transient B-cells and is irreversibly lost by all memory B-cells [131]. At the same time, ABCB1 expression can affect the migration ability and location of naive B-cells relative to transient B-cells and memory B-cells, as well as influence the structure and function of lipid rafts, which are crucial for B-cell signaling. Lipid rafts act as platforms in B cells for B-cell receptor (BCR) signaling and antigen targeting, and the association of BCRs with lipid rafts changes during B-cell development [132].

In addition, ABCB1 can participate in the pathogenesis of various skin diseases [133]. A protective role of these transporters has been shown in a model of dermatitis in *Mdr1a/1b/Bcrp*^−/−^ mice, as lesions in *Mdr1a/1b/Bcrp*^−/−^ mice were more severe [134]. On the other hand, ABCB1 inhibitors are considered as a candidate for a possible therapeutic agent for the treatment of acne. Propionibacterium acnes has been shown to promote sebum secretion due to ABCB1 activation simultaneously with increased ABCB1 expression, which may be the result of TLR2 pathway activation in differentiated hamster sebocytes (DHS) [135]. Uveal melanoma cells expressing ABCB1 have also been shown to have a greater potential for metastasis, while exhibiting significantly increased mitochondrial activity compared with *ABCB1*^−/−^ [136].

Numerous studies have also demonstrated the importance of ABCB1 in the pathogenesis of Alzheimers disease [137,138,139,140,141,142]. Amyloid beta (Aβ) deposition in Alzheimer’s disease has been shown to be inversely correlated with ABCB1 expression in the brains of elderly people without dementia, which may indicate that age-related ABCB1 deficiency may be involved in the pathogenesis of Alzheimer’s disease [138,143]. Indeed, normal aging is characterized by a progressive decrease in ABCB1 activity in the blood-brain barrier [144,145]. In this case, ABCB1 is involved in Aβ transport, while disruption of this process may be associated with the development of Alzheimer’s disease [137,138,139,140,141,142].

Thus, ABCB1, in addition to the transport of xenobiotics, is involved in the immune defense of the body, regulating a number of processes in both cells of the innate and adaptive immune systems. These and other data suggest that ABCB1 is a promising target for drug interventions, which are mainly aimed at overcoming multidrug resistance in cancer.

### 3.3. The ABCC Subfamily

Members of the ABCC subfamily, such as ABCC1 (MRP1), ABCC2 (MRP2), ABCC3 (MRP3), ABCC4 (MRP4), ABCC5 (MRP5), ABCC6 (MRP6), and ABCC10 (MRP7), are known for their roles in multidrug resistance mechanisms [146]. In addition, MRP1 [147], MRP2 [148], MRP3 [149], MRP4 [150], MRP6 [151], MRP7 [152], and MRP8 [153] are involved in LTC 4 transport [154]. MRP1 is also involved in the transport of leukotriene D4 (LTD4) and LTE 4, which are metabolized forms of LTC 4 [147]. *MRP1*^−/−^ mice have shown an impaired response to inflammatory stimuli, which is associated with decreased LTC 4 secretion [155,156]. ABCC4 also mediates ATP-dependent outflow of LTB 4, which is a hydrolyzed form of the LTA 4 precursor [150,154]. Given that leukotrienes are lipid mediators involved in inflammation, the transport activity of ABCC may be involved in the regulation of inflammation. It has been shown that *mrp1*^−/−^ mice were resistant to pneumococcal pneumonia, through the increased release of LTB4, which mediates enhanced bacterial clearance in these mice. Increased LTB 4 production is associated with intracellular accumulation of LTC4, a product inhibition of LTC 4 synthase that eliminates substrate competition between LTC4 synthase and LTA4 hydrolase for substrate [157].

ABCC1 is also involved in the export of Sphingosine-1-phosphate (S1P), a lipid mediator involved in many biological processes, including inflammation, angiogenesis, apoptosis, macrophage function, and regulation of endothelial barrier integrity.

In addition, the human multidrug resistance protein MRP4 actively transports the prostaglandins PGE 1 and PGE 2 [156]. Through participation in the transport of PGE 2, MRP4 significantly contributes the migration of human dendritic cells to the draining lymph nodes, which is important for the initiation of the immune response [158,159,160,161]. Inhibition of MRP4 has been shown to reduce the number of migrated skin dendritic cells by 60–70% [161,162,163]. ABCC4 is also involved in the regulation of fibroblast migration. In this process, F-actin was identified as a downstream target and the main mediator of MRP4 effect on cell migration, with cAMP/cGMP playing the role of signaling molecules [163,164].

The role of MRP1 in adaptive immunity is of interest. Adaptive immune responses begin after the transfer of antigen-containing dendritic cells from peripheral tissues to the lymph nodes. In doing so, MRP-1 regulates the migration of dendritic cells into the lymph nodes by transporting LTC4, which promotes chemotaxis to CCL19 and mobilization of dendritic cells from the epidermis [165].

Another member of this subfamily, MRP2 regulates mucosal inflammation by enhancing neutrophil transmigration, which is associated with increased hepoxylin A3 (HXA 3) synthesis [124]. HXA 3 is a biologically relevant eicosanoid formed from the intermediate 12S-hydroperoxy-5Z, 8Z, 10 E, 14 Z-eicosatetraenoic acid (12 S-HpETE) formed in the 12-lipoxygenase pathway of arachidonic acid metabolism through hepoxylin synthase activity [124,166,167,168,169].

### 3.4. The ABCG Subfamily

Many members of the ABCG subfamily are known for their role in lipid homeostasis, among which ABCG1- and ABCG4-transporters, which are half ABC proteins, are the best characterized. They consist of one transmembrane domain and one nucleotide-binding domain, but for activation, the proteins form a dimer (homodimer or heterodimer) or even an oligomer depending on function [47,170,171,172].

ABCG1 is expressed in many cell types, including myeloid cells, lymphocytes, epithelial and endothelial cells of various organs, and transports cholesterol, 7-ketocholesterol, sphingomyelin, and phosphatidylcholine from cells to HDL [171,173,174]. ABCG1, along with ABCA1, protects cells from sterol overload by removing its export from peripheral cells and saturating them with HDL [172,175,176]. ABCA1 is thought to provide initial cholesterol saturation of lipid-poor apoA-I, thus forming “nascent” HDLs, and then ABCG1 further saturates them with cholesterol [177,178,179,180,181].

ABCG1 is also involved in the regulation of inflammation. Macrophages from *Abcg1*^−/−^ mice have increased inflammatory activity and had lipid accumulation [182]. Increased levels of the inflammatory cytokines IL-6, IL-1β, IL-1α, and IL-12, and decreased levels of the anti-inflammatory IL-10 in alveolar macrophage cell supernatants were shown. In addition, there was an increase in monocyte recruitment and macrophage differentiation in the lungs of *Abcg1*^−/−^ mice and increased apoptosis of alveolar macrophages, which generally did not lead to a change in lung macrophage content [182]. At the same time, *Abcg1*^−/−^ macrophages engulfed many apoptotic cells but could not fully excrete cholesterol derived from these apoptotic cells, which is consistent with the propensity of *Abcg1*^−/−^ alveolar macrophages for apoptosis [182].

ABCG1 has also been shown to be involved in macrophage polarization. ABCG1 deficiency contributes to proinflammatory M1 polarization of human macrophages. This mechanism is thought to be mediated through the Akt signaling pathway [183]. Another study showed that increased cholesterol accumulation in macrophages in the absence of ABCG1 causes NF-κB activation, which polarizes these macrophages to a proinflammatory M1 phenotype [184]. It has also been shown that ABCG1 deficiency is sufficient to inhibit the chemotaxis of M2 macrophages [27].

Thus, changes in cholesterol homeostasis due to lack of ABCG1 modulate immune cell function. These and other data identified an important role of ABCG1 in the prevention of atherosclerosis, which allows us to consider ABCG1 along with ABCA1 as potential therapeutic targets for the prevention of atherosclerosis through the regulation of their expression, localization, and degradation [185].

## 4. Conclusions

**Figure 3:**
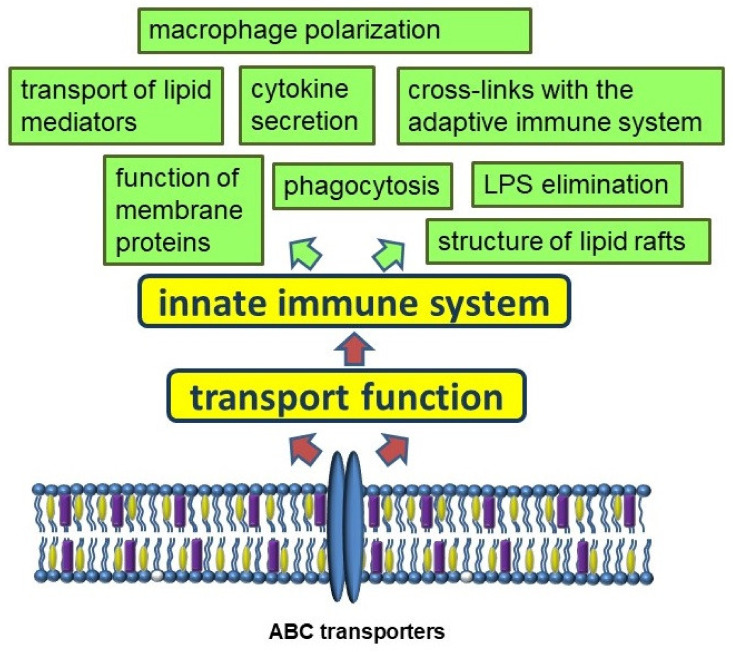
Participation of ABC transporters in the innate immune system.

A growing body of evidence strengthens the understanding of the importance of ABC transporters in immune function (Figure 3). The links between lipid metabolism and the innate immune system are of interest. Among a large family, about 20 members are involved in lipid transport. The lipid-transporting activity of ABC transporters results in the regulation of plasma membrane composition and function, LPS utilization and response, and transport of some lipid mediators involved in inflammation. In addition, ABC transporters are involved in the mechanisms of export of xenobiotics, as well as in the regulation of the links between the innate and adaptive immune systems.

These and other data allow us to consider ABC transporters as an important promising target for drug action. This is considered most relevant for overcoming the mechanisms of multidrug resistance to chemotherapeutic agents, which is important in oncology. In this regard, the ABCB1 transporter is the object of close attention of clinicians and researchers.

Importantly, there is a growing body of evidence supporting cross-linkages between multidrug resistance mechanisms and the host immune defenses. In addition, data on the role of ABC transporters in the relationship between the human body and the microbiota are of interest. A growing body of evidence strengthens the understanding of its role in the immune system, the regulation of the function of various organs, such as the lungs. In this regard, given some evolutionarily conserved relationships in the structure and function of ABC transporters, both the involvement of bacteria in the regulation of human ABC transporters and the role of bacterial ABC transporters in impairment of the normal gut microbiome appear of interest. Understanding these mechanisms can be useful in analyzing the effectiveness of drug therapy.

The regulation of the functional activity of ABCA1 and ABCG1 and their disorders are also of clinical interest and are a promising target for the treatment of atherosclerosis. At the same time, a large number of data confirm the role of these transporters in the immune system, which is a promising topic for future research.

Thus, ABC transporters demonstrate various physiological functions, including in providing immune protection to the body. A better understanding of these functions will improve the diagnosis and treatment of many diseases.

## Data Availability

Not applicable.
